# Nontuberculosis mycobacteria (NTM) infections in patients with leukemia: a single center case series

**DOI:** 10.3389/fmed.2024.1402897

**Published:** 2024-08-01

**Authors:** Jennifer Marvin-Peek, Koji Sasaki, Dimitrios P. Kontoyiannis, Javier Adachi, Maro Ohanian, Koichi Takahashi, Ghayas C. Issa, Steven Kornblau, Hussein A. Abbas

**Affiliations:** ^1^Department of Cancer Medicine, The University of Texas MD Anderson Cancer Center, Houston, TX, United States; ^2^Department of Leukemia, The University of Texas MD Anderson Cancer Center, Houston, TX, United States; ^3^Department of Infectious Disease, The University of Texas MD Anderson Cancer Center, Houston, TX, United States; ^4^Department of Genomic Medicine, The University of Texas MD Anderson Cancer Center, Houston, TX, United States

**Keywords:** AML, nontubercolous mycobacteria, NTM, leukemia, ALL

## Abstract

Patients with leukemia experience profound immunosuppression both from their underlying disease as well as chemotherapeutic treatment. Little is known about the prevalence and clinical presentation of nontuberculous mycobacteria (NTM) in this patient population. We identified six cases of NTM infection from 29,743 leukemia patients who had acid-fast bacilli (AFB) cultures. Four cases had bloodstream infections and five had disseminated disease, including one who presented with an unusual case of diffuse cellulitis/myositis. All patients were lymphopenic at time of diagnosis, and two patients ultimately died from their NTM infection. NTM infections are a rare, but potentially life-threatening infection in patients with leukemia. Sending AFB cultures early is important to direct appropriate antimicrobial therapy and allow for future leukemia-directed therapy.

## Background

Leukemias are diverse blood cancers marked by the rapid growth of abnormal blood cells, causing bone marrow failure and systemic complications (1). Importantly, infections are a leading cause of death in leukemia patients due to their weakened immune systems and the immunosuppressive effects of chemotherapy (2). The risk of severe viral, bacterial, and fungal infections is well described, and therefore it is typical for patients with leukemia to receive antimicrobial prophylaxis against these standard pathogens (3). However, less is known about the risk of nontuberculous mycobacteria (NTM) and its presentation in patients with leukemia. NTM are a diverse group of bacteria that are ubiquitous in the environment, often present in soil and water reservoirs (4). NTM are typically classified into either rapid growing mycobacteria (RGM) or slowly growing mycobacteria (SGM) based on time for mature colony formation in solid growth medium, with *Mycobacterium abscessus* and *Mycobacterium avium-intracellulare complex* (MAC) being most common subtypes, respectively (5, 6). With >200 NTM species identified to date, several meta-analyses suggest that the prevalence of NTM infection is increasing across the globe (7, 8). Multiple host and environmental factors are hypothesized to contribute to this rise including the increasing age of the general population, higher prevalence of chronic lung disease, greater use of immunosuppressive medications, and increasing vapor pressor (i.e., warm, humid environments) (9, 10). Indwelling lines are an additional known risk factor for disseminated NTM, and previous case reports and series suggest that procedures where the skin barrier is breached (e.g., acupuncture, surgical procedures, or trauma) may precede NTM skin infections in in immunocompetent hosts (11–15).

Nonetheless, NTM infections are generally uncommon in immunocompetent hosts as normal immune defense mechanisms are often able to prevent symptomatic infection. Typically, mycobacteria are phagocytosed by macrophages, which secrete IL-12 and IFNγ to recruit CD4 T-cells to assist in macrophage activation and killing of intracellular pathogens (6). The activated macrophage also produces TNFα to recruit neighboring T and B-cells, resulting in the formation of a granuloma to contain the mycobacterial infection. Patients with advanced HIV or genetic syndromes with germline IL-12 and IFNγ mutations often experience a greater incidence of NTM infections (16). Given the demonstrated importance of cellular immunity in mycobacterial control, other populations with impaired immune systems, such as patients with leukemias, may also be more susceptible to symptomatic or disseminated NTM disease. Previously published case series report a higher incidence of mycobacterial infection in patients with cancer compared to the general population (17, 18), although they include only a limited number of patients with hematologic malignancies. A recent meta-analysis of NTM infection after allogenic stem cell transplant (SCT) suggests that the rate of NTM infection is also significantly higher in SCT patients than in healthy individuals (19). In a retrospective dataset of 118 patients hospitalized with NTM infection in China, patients who were immunocompromised (*n* = 64) had a significantly high mortality than those were not (*n* = 54; HR 3.537, 95% CI 1.526–8.362), highlighting the importance of better characterizing this population (20).

The prevalence and clinical characteristics of NTM infections among leukemia patients, who are severely immunodeficient, is not described before. Herein, we report our single center experience of six patients diagnosed with leukemia who developed NTM infection to provide insight into predisposing factors and possible presentations in this unique population.

## Methods

NTM infections were identified by positive acid-fast bacilli (AFB) cultures from January 4, 2016–November 30, 2023 in patients admitted with a diagnosis of leukemia to the University of Texas MD Anderson Cancer Center in Houston, TX. The identification of and mycobacteria speciation was done using 16S ribosomal DNA sequencing. As previously described, this was done using a polymerase chain reaction (PCR) followed by gene sequencing of the first 500–600 bp of the 16S gene to identify the NTM species (21). ATS/IDSA clinical practice guidelines were used to distinguish NTM disease from colonization and/or environmental contamination (16). Patients needed meet both clinical (symptoms and/or nodular or cavitary opacities on radiography) and microbiologic criteria [two positive AFB sputum cultures, positive bronchoalveolar lavage (BAL) culture, biopsy showing mycobacterial histologic features, or positive blood culture] to have true mycobacteria infection. Disseminated infection was defined as either multiple cutaneous abscesses, visceral involvement, or positive blood cultures as previously described (17). To detect somatic mutations in bone marrow specimens, sequencing libraries were prepared from DNA using Agilent Haloplex-based target enrichment of the genomic regions of interest in 81 genes. Bidirectional paired-end sequencing was performed using an Illumina MiSeq (for patients diagnosed prior to 2022) or NextSeq platform (for patients diagnosed after 2022) to screen for single nucleotide variants and insertions/deletions up to 52 base-pairs. The genomic reference sequence used is gnome GRCh37/hg19. Clinical data was gathered via retrospective chart reviews. This case series was approved by the Institutional Review Board.

## Results

There were 29,743 patients with leukemia seen at MD Anderson Cancer Center who had AFB cultures collected from 2016 to 2023. Cultures were most often drawn for persistent fevers or pulmonary infiltrates concerning for possible NTM. Eight patients had at least one positive culture. Two patients with positive BAL cultures (one with *M. avium* and the other with *M. gordonae*), were deemed not to have true infection due to lack of concordant radiographic or any pulmonary symptoms. Both patients were evaluated by infectious disease specialists on multiple visits and the positive cultures were thought to be due to colonization and culture contamination, respectively, and were not treated with any NTM-directed therapy.

From the six patients with true NTM infection (incidence of 0.02%), four were male and two were female with a median age of 71 years (range 28–81 years; Table 1). Three patients had acute myeloid leukemia (AML), two myelodysplastic syndrome (MDS), and one B-cell acute lymphoblastic lymphoma (ALL) diagnosed at median 17.3 months prior to NTM infection (range 1.3–28.5 months). Two had patients had undergone previous allogeneic stem cell transplant (SCT). Five of the six patients were undergoing active chemotherapy at time of their NTM diagnosis. The one patient who was not receiving chemotherapy had been in a complete remission (CR) since their SCT 11.9 months prior.

**Table 1:** Patient demographics, clinical characteristics, and outcomes of NTM infection.

	Case number					
Variables	1	2	3	4	5	6
Age (years), sex	78, F	53, M	81, M	70, F	28, M	72, M
Comorbidities	HTN	CKD, recent *Rhizopus* PNA	HTN, recent *Pseudomonas* and MSSA PNA	HTN	Recent typhlitis with hemicolectomy	Rheumatoid arthritis, DM2, HTN
Leukemia diagnosis	B-ALL	AML	AML	MDS	AML	MDS
Mo. from leukemia diagnosis*	11.4	18.6	1.3	28.5	16	26
Leukemia status	Relapsed	CR^†^	PR	Persistent	Relapsed	Persistent
Active chemo*	Yes	No	Yes	Yes	Yes	Yes
Stem cell transplant	No	Yes	No	No	Yes x2	No
Mo. from SCT*	N/A	11.9	N/A	N/A	11.0, 3.5	N/A
NTM species isolated	*M. canariasense*	*M. fortuitum*	*M. abscessus*	*M. intracellulare*	*M. abscessus*	*M. abscessus*
Culture site	Blood	BAL	BAL + Blood	BAL + Blood	SSTI+Blood	SSTI
Days for culture positivity	3.8	17	9	23	3.7	16
Disseminated	Yes	No	Yes	Yes	Yes	Yes
Reason for AFB culture	Neutropenic fever	Hypoxia requiring intubation; pulmonary infiltrates	Pulmonary infiltrates; cough	Neutropenic fever; pulmonary infiltrates	Neutropenic fever	Fever + skin/soft tissue lesions
Antibiotic prophylaxis	Cefpodoxime	Azithromycin	Augmentin	Levofloxacin	Levofloxacin	Levofloxacin
NTM sensitivities	S = CFX, IMI, MOX, CLA, AMI, TOB, DOX, TIG, TMP/SMX, LIN I = CIP R = None	N.D.	S = AMI, CLO, TIG I = IMI, CFX R = CLA	S = CLA, AMI I = None R = LIN, MOX, CLO	S = AMI I = CFX, IMI R = CIP, MOX, CLA, TOB, DOX, TMP/SMX, LIN	N.D.
NTM treatment	AZI + IMI + LIN	N/A (dx after death)	AMI + CLO + TIG + IMI-CIL	N/A (dx after death)	AMI + AZI + TIG + IMI-CIL	AZI + IMI-CIL + LIN
Additional notes	N/A	Concurrent coronavirus, influenza A H3, RSV	N/A	Concurrent EBV viremia	Concurrent *E. faecalis*, *S. epidermidis*, *S. salivarius* bacteremia, and *C. glabrata* fungemia	N/A
Outcome at 30 days	Cleared NTM blood Cx	Death 17 days after positive culture	Cleared NTM blood Cx, improvement in CT infiltrates	Death 16 days after positive culture	Cleared NTM blood Cx, death from fungemia	Improvement in skin and soft tissue lesions

*At time of positive mycobacterial culture. ^†^With negative MRD. AFB, acid fast bacilli; AML, acute myeloid leukemia; BAL, bronchoalveolar lavage; B-ALL, B-cell acute lymphoblastic leukemia; HTN, hypertension; CKD, chronic kidney disease; CR, complete remission; DM2, type II diabetes mellitus; MDS, myelodysplastic syndrome; MSSA, methicillin sensitive *Staphylococcus aureus*; NTM, nontuberculous mycobacteria; PNA, pneumonia; PR, partial response; SSTI, skin and soft tissue infection. Antibiotics: AMI, amikacin; AZI, azithromycin; CFX, Cefoxitin; CIL, cilastatin; CIP, ciprofloxacin; CLA, clarithromycin; CLO, clofazimine; DOX, doxycycline; IMI, imipenem; LIN, linezolid; MOX, moxifloxacin; TIG, tigecycline; TMP/SMX, trimethoprim/sulfamethoxazole; TOB, tobramycin.

The NTM species cultured were *M. abscessus*, *M. canariasense, M. fortuitum,* and *M. intracellulare*. All species isolated except for *M. intracellulare* are classified as rapidly-growing mycobacteria (RGM). NTM were cultured from the blood in four cases, BAL in three, and skin-soft tissue (SSTI) in two with a median of 9 days (range 3.7–17.0 days) to positive culture for RGM (*n* = 5) or 23 days for SGM (*n* = 1). Disease was disseminated in five of the six patients. The one case of pulmonary-limited disease (Case #2) was in the only patient not undergoing active chemotherapy at NTM diagnosis. This patient was in a CR and no longer had an indwelling central venous catheter (CVC) unlike the other five patients. Case #6, interestingly, presented with only disseminated non-specific skin lesions/nodules with a persistent cellulitis/myositis for which skin biopsies ultimately grew *M. abscessus.* They were also the one patient who did not have an indwelling central venous catheter (CVC) at NTM diagnosis. Three patients were diagnosed with concurrent infections including non-COVID-19 Coronavirus, *Influenza* A, polymicrobial bacteremia, and fungemia. Four of six patients were neutropenic at time of positive culture (ANC range: 0–7,180/μL), but all were lymphopenic (ALC range: 0–550/μL; Table 2). No patients had received prior alemtuzumab or were on steroids at time of diagnosis.

**Table 2:** Disease characteristics of leukemia in patients diagnosed with NTM.

	Case number					
Variables	1	2	3	4	5	6
NTM species isolated	***M. canariasense***	***M. fortuitum***	***M. abscessus***	***M. intracellulare***	***M. abscesses***	***M. abscessus***
Leukemia diagnosis	B-ALL	AML	AML	MDS	AML	MDS
WBC (K/μL)*	0	2.3	0.3	0.7	0	9.2
ANC (no./μL)*	0	1850	20	450	0	7,180
ALC (no./μL)*	0	210	260	10	0	550
AMC (no./μL)*	0	180	20	60	0	90
Hemoglobin (g/dL)*	9	7.9	9.1	7.8	8.6	8
Platelets (K/μL)*	19	39	115	9	17	94
Peripheral blast %*	0	0	2	1	0	1
Cytogenetics	46,XX[13] / 50-52,XX,+X,+1,+3,+6,+6,add(6)(q24),add(7)(q32),add(18)(q23),+12mar[cp7]	48,idem,+19[17] / 47,XY,+8,t(8;9)(p11.2;q33)[3] (pre-SCT) Diploid (post-SCT)	44,X,-Y,-20[1] / 4,647,idem,+der(2;17)(p10;q10)[cp4] / 45,XY,der(2;17)(p10;q10),+del(11)(q13q14),-12,-16,add(17)(q11.2)[12]	46,XX,+1,der(1;7)(q10;p10)[20]	48,XY,t(6;11)(q27;q23),+22[20]	Diploid
FISH	*KMT2A* (*MLL*) rearrangement	Negative	Negative	Negative	*KMT2A* (*MLL*) rearrangement	Negative
NGS mutations	*KMT2A, TP53*	*RUNX1*	*BCOR, DNMT3A, TET2, TP53*	*SF3B1*	*DDX41, GATA2, CREBBP, RAD21*	*ASXL1, ETV6, EZH2, IKZF1, JAK2, SETBP1, ZRSR2*
Current therapy	SNDX-5613 + FLA	None	Decitabine + BP1001 + VEN	AZA + VEN	Cladribine + LDAC + VEN	AZA + VEN
No. of lines of therapy	4	1	1	5	3	5

*At time of positive mycobacterial culture. ALC, absolute lymphocyte count; AMC, absolute monocyte count; ANC, absolute neutrophil count; AML, acute myeloid leukemia; AZA, azacitadine; B-ALL, B-cell acute lymphoblastic leukemia; BP1001, liposomal Grb2 antisense oligonucleotide; FISH, fluorescence in situ hybridization; LDAC, low dose ara-C (cytarabine); NGS, next generation sequencing; MDS, myelodysplastic syndrome; NTM, nontuberculous mycobacteria; SNDX-5613, menin inhibitor; VEN, venetoclax; WBC, white blood cell.

Because the diagnosis of NTM was reached after two patients had passed from infection, only four patients received treatment for NTM with either three or four-drug antimicrobial regimens. All four had combinations including a macrolide and imipenem. The three patients who received treatment who had NTM bacteremia cleared their cultures, and all four clinically improved from their infections. All subsequently resumed chemotherapy for their underlying leukemia, and one patient is currently undergoing hematopoietic stem cell transplant.

The cytogenetics and molecular characteristics of each cases’ underlying leukemia are detailed in Table 2. Of patients with AML as their primary leukemic diagnosis, two were *de novo* and one was secondary from prior myeloproliferative neoplasm. Of note, Case #5 did have a GATA2 mutation with an allele frequency of 44%. While germline GATA2 mutations have been associated with “MonoMAC syndrome,” this mutation was not present on previous bone marrow biopsies suggesting that this was not a germline event.

## Discussion

We report six confirmed cases of NTM infection in adult patients with leukemia at a large academic cancer center over an eight-year period. This represents an incidence rate of 0.02% in leukemia patients with AFB cultures. Other studies have reported higher rates of NTM infection than our single-center experience. A retrospective study from 2001 to 2020 published from Taiwan cited an incidence of 1.2% in patients with all types of hematologic malignancy, noting that neutropenia was the greatest predictor of infection (22). An older case series of patients with hairy cell leukemia in 1986 reported an incidence of 5% (9/186) (23). The rarity of NTM infection in patients with leukemia in our single-institution experience could reflect differences in NTM prevalence across the globe, differences in standard antimicrobial prophylaxis, and/or changes in sanitation and sterilization procedures over time. Over the last year, many of the published case reports of NTM infection in patients with leukemia predominate in pediatric patients or patients who have undergone SCT (19, 24–29). While we unfortunately are not able to capture pediatric patients in our case series, two of our six cases were in patients with prior SCT. The high incidence of NTM infection in patients with prior allogenic SCT is thought to be due to prolonged impaired cell-mediated immunity from conditioning regimens containing anti-thymocyte globulin as well as immunosuppressive medications for prevention of graft-versus-host-disease (GVHD). Patients frequently receive antibiotics for *Pneumocystis jiroveci* pneumonia prophylaxis while taking GVHD preventative medications, but their antimicrobial spectrum often does not cover NTM.

While the incidence of NTM infection was quite rare in our cohort, it was still higher than estimated in the general population (7), which is likely reflective of the degree of immunosuppression patients with leukemia experience. Three of the six patients in this case series had concurrent infections and five had disseminated disease, supporting how profoundly immune suppressed this patient population is. Neutropenia, although common, was not universal in this case series. All six patients, however, were lymphopenic. Immune subset studies were not performed in this study but given the known importance of CD4 T-lymphocytes in control and prevention of disseminated NTM infection, we suspect low T-lymphocyte abundance may have been an important predisposing factor.

In addition to their immune suppression, patients with leukemia often have indwelling central venous catheters (CVCs) which are necessary for delivery of chemotherapy and frequent blood transfusions, but can also serve as a nidus for infection. In this case series, all patients with bloodstream NTM infection had a CVC. Other case series have reported a similarly high presence of CVCs in cancer patients diagnosed with NTM bacteremia (at 97%) (17), particularly with *M. canariasense* which has only been described as catheter-related (30, 31). All patients in this series that had NTM bacteremia and positive blood cultures had their CVC removed, including case #1 with presumed line-associated *M. canariasense*. Unfortunately, none of the patients had the catheter tips saved for culture to confirm line association. While it is unclear if the CVC was the initial source or just became colonized, regardless this highlights the importance of using sterile technique and saline for cleansing and dressing CVCs in this patient population.

One patient in this case series also possessed a *GATA2* mutation. Autosomal dominant deficiencies in *GATA2* have been associated with a “MonoMAC” syndrome, in which patients exhibit a deficiency of mononuclear phagocytes, NK, and B-cells (32). In addition to possessing a high risk of leukemia/myelodysplasia, they are also predisposed to NTM infections due to abnormal regulation of hematopoiesis. Although this patient did not have a germline *GATA2* mutation based on prior bone marrow biopsies, it is possible that acquiring this mutation during leukemic progression may contribute to immune dysregulation with increased predisposition to disseminated NTM infection. While the number of cases is limited in this report, larger studies investigating how genetic changes in leukemia may pre-dispose to opportunistic infections such as NTM may be of clinical value.

Ultimately, two patients died from NTM in this case series. In Case #4 with *M. intracellulare* in the blood and BAL cultures, her AFB cultures took 23 days to result which can be quite common with slowly-growing mycobacteria. Unfortunately, she died prior to the culture result and never received NTM directed therapy. While Case #2 had *M. fortuitum* which is typically a rapid-growing mycobacteria, BAL cultures still took 17 days to grow and by then the patient had passed from refractory hypoxemia. However, his case was complicated by multiple other pulmonary infections including respiratory syncytial virus (RSV), coronavirus, influenza, and *actinomyces* which may have also contributed to his respiratory failure. There is currently no published data that other infections can predispose to NTM infection, but certainly chronic lung abnormalities such as bronchiectasis, cystic fibrosis, and COPD have been associated in healthy individuals (16). Maintaining a high index of clinical suspicion for NTM in patients with leukemia given their level of immune compromise is critical such that AFB cultures are sent in a timely fashion and they can be treated accordingly. Given that AFB cultures are not routinely sent for neutropenic fever, it is possible that NTM infections are underdiagnosed in this population. Prospective studies evaluating the utility of sending AFB cultures for leukemia patients with persistent neutropenic fever. The other four patients in this case series fortunately had early AFB cultures sent and were able to be started on treatment and recovered from their illness. All four received subsequent chemotherapy without obvious relapse of their infection, and one is currently undergoing SCT.

Metagenomic next-generation sequencing (mNGS) is a newer technology that identifies pathogens by amplifying all sequencing all DNA in samples (33). An advantage of mNGS is that it provides an unbiased approach (compared to PCR and primer-based methods) and could identify more rare and slow-growing organisms such as NTM more quickly (34). However, a concern is that such a sensitive technique may not be able to easily distinguish between physiologic colonization versus true pathogenic infection. Furthermore, other studies have shown difficulties detecting *M. tuberculosis* in the background of biological/colonizing NTM (35). While this type of unbiased and rapid pathogen identification is promising, before to mNGS being used in clinical practice for leukemia patients, it would need to be validated in NTM and in an immune compromised population.

In summary, this case series details six cases of NTM infection in patients with leukemia out of 29,743 AFB cultures sent in over a 7-year time period. All patients were lymphopenic at time of positive culture, and most had disseminated disease. Although NTM infection was extremely rare with an incidence of 0.02%, two of six patients died from NTM in this series (33%). In both cases, NTM directed therapy was never initiated as their AFB cultures required >2 weeks to grow. Given the high mortality of disseminated NTM infection, clinicians should consider sending early AFB cultures in patients with leukemia who experience neutropenic fevers, abnormal pulmonary nodules, or unusual skin lesions as proper antimicrobial treatment could allow for successful leukemia-directed therapy in the future.

## Data availability statement

All original contributions to the data are presented in the article. For patient confidentiality and participant privacy, additional requests for data should be directed to the corresponding author.

## Ethics statement

The studies involving humans were approved by MD Anderson Cancer Center Institutional Review Board. The studies were conducted in accordance with the local legislation and institutional requirements. Written informed consent for participation was not required from the participants or the participants’ legal guardians/next of kin in accordance with the national legislation and institutional requirements.

## Author contributions

JM-P: Data curation, Writing – original draft, Writing – review & editing. KS: Data curation, Methodology, Writing – review & editing. DK: Writing – review & editing. JA: Writing – review & editing. MO: Resources, Writing – review & editing. KT: Resources, Writing – review & editing. GI: Resources, Writing – review & editing. SK: Resources, Writing – review & editing. HA: Conceptualization, Methodology, Resources, Supervision, Writing – review & editing.
